# Prognostic significance of neutrophil-lymphocyteratio/platelet-lymphocyteratioin lung cancers: a meta-analysis

**DOI:** 10.18632/oncotarget.12526

**Published:** 2016-10-08

**Authors:** Hong-Bo Yang, Meng Xing, Lei-Na Ma, Ling-Xin Feng, Zhuang Yu

**Affiliations:** ^1^ Oncology Department, The Affiliated Hospital of Qingdao University, Qingdao, People's Republic of China

**Keywords:** NLR, PLR, OS, clinicopathologic parameters, lung cancers

## Abstract

**Setting:**

For now, hematological markers of inflammatory response have emerged as prognostic factors for patients with cancer. Many articles have confirm that neutrophil to lymphocyte ratio(NLR) and platelet–lymphocyte ratio (PLR) are relate with poor prognosis in various types of tumors.

**Objective:**

To investigate the association between NLR/PLR and progression free survival (PFS), overall survival (OS) and clinicopathologic parameters in lung cancer patients.

**Design:**

We performed relevant searches in PubMed database, Google Scholar, Springer Link. We included retrospective cohort studies that reported hazard ratios with 95% confidence intervals for the NLR or PLR and PFS or OS.

**Results:**

Both high NLR (*P* < 0.00001) and high PLR (*P* = 0.01) were significantly predictive of poorer OS. It also demonstrated that elevated NLR predicted poorer PFS (*P* = 0.0002). High NLR was significantly associated with deeper Invasive of tumor, (*P* = 0.006) extensive lymph nodetastasis(N2–3) (*P* = 0.01), poor differentiation (*P* = 0.0002) and vascular invasion(*P* = 0.002). There was no evidence of publication bias. Subgroup analysis indicated that little evidence of heterogeneity. However, PLR has no prognostic significance for SCLC.

**Conclusions:**

We provides further evidence in support of elevated NLR and PLR were predictors of poor OS and PFS in patients with lung cancer. Given this, NLR and PLR may be markers to report treatment outcomes.

## INTRODUCTION

It is widely believed that systemic inflammatory response is important in monitoring tumor progression and evaluating prognosis in many cancer types and then influence survival outcomes in cancer patients. Many hematological parameters such as neutrophil counts [1], monocyte counts [2], platelet counts [3], which are as components of systemic inflammation factors, neutrophil-lymphocyte ratio (NLR) [4] and platelet lymphocyte ratio [5], have proved to be indicators that have prognostic implications with many types of cancer in many studies. As we all known, Neutrophil–lymphocyte ratio (NLR) can easily get and is an important sign of inflammatory processes. The relevance between high NLR and poor prognosis in many types of cancers such as the breast cancer, kidney cancer, pancreas cancer, and stomach cancer be reported in many researches [1, 6]. Platelet to lymphocyte ratio (PLR), another factor which exerts a very important effect on the pathogenesis of systemic inflammatory response, was also proved to be associated with survival in patients with cancer [7]. Several studies have established that the platelet is supplementary of the role of neutrophils in the blood vessel metastasis [8]. However, the prognostic role of NLR and PLR in lung cancer and their significance in the clinical and pathological features still needs more studies to prove. Given the newly emerging evidence, we conducted a meta-analysis of retrospective cohort studies with the following objectives:(1):to systematically review, summarize and further confirm the prognostic value of NLR and PLR in lung cancer patients(2):to evaluate the impact of NLR and PLR on clinical and pathological features parameters of lung cancer.

## RESULTS

### Search outcomes

**Figure 1:**
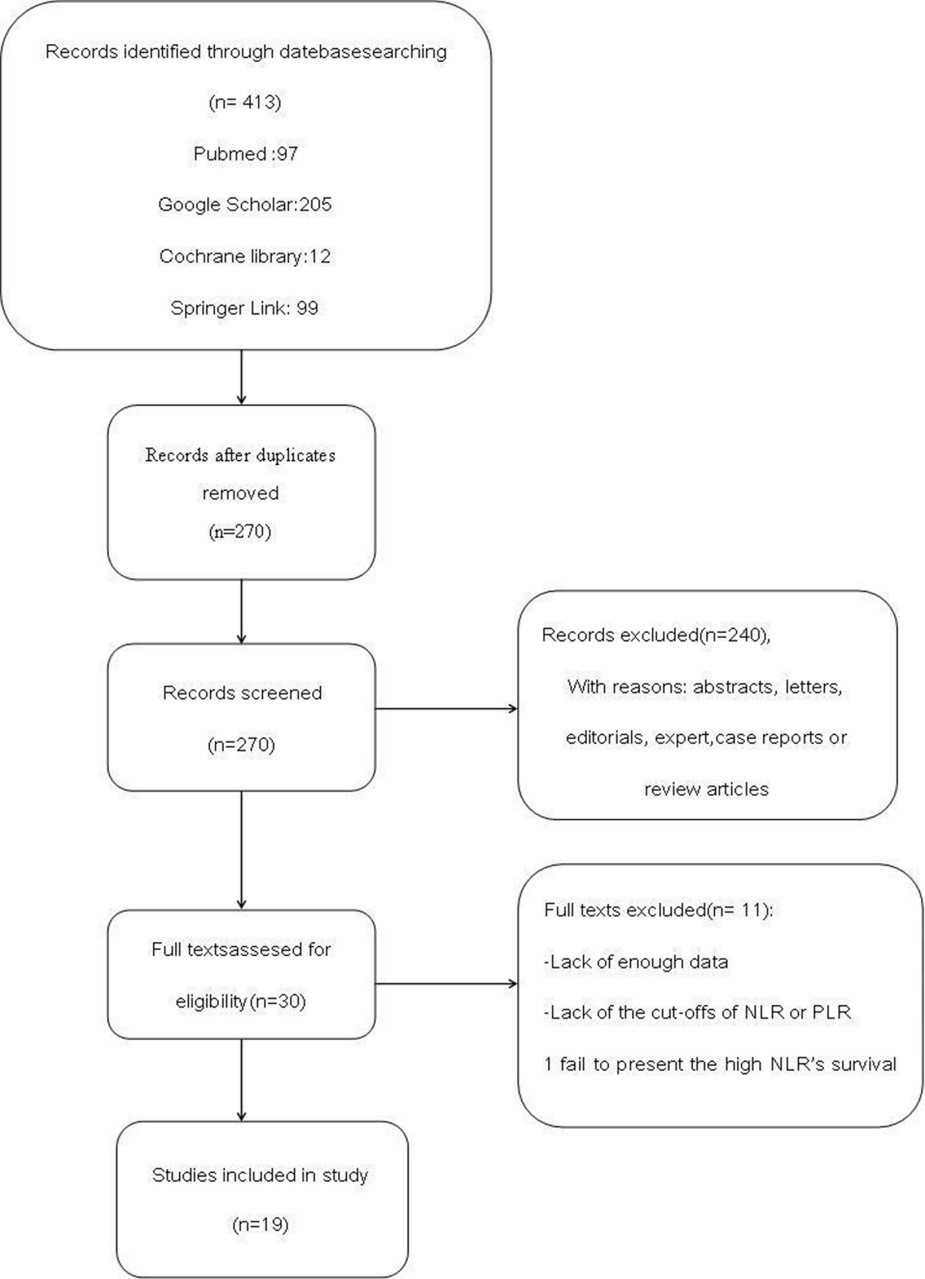
Flow chart demonstrating process of study selection

Nineteen studies involving 7,283 patients were included in the meta-analysis. A flow chart showing the study selection is presented in Figure 1. We initially retrieved unique studies. Of these, 143 citations were excluded after duplicated data leaving, 240 citations are left after screening based on abstracts or titles. Of these studies, 10 citations were excluded because of lack of enough data or the cut-offs of NLR or PLR, one citation fail to present the high NLR's survival data. Thus leaving 19 studies for the final analysis. Characteristics of the selected studies are presented in Table 1. All 19 retrospective cohort studies were published between 2012 and 2015. Among them, Nine studies were conducted in the China, three in Turkey, two in Japan, two in Korea, and one in Spain, one in UK, one in USA. The number of participants ranged from81 to 1,238, with a sum of 7,283, including 5,881 with NSCLC1,402 with SCLC. Seven of the studies evaluated both NLR and PLR. The other twelve evaluated only NLR. The cut-off value for HNLR was < 3 in seven studies, 3 ≤ to < 5 in six studies and ≥ 5 in five study. ALL studies evaluated OS outcomes. Eight studies evaluated both OS and PFS outcomes. In eighteen of the enrolled cohorts, HRs and 95% CIs reported directly. All of nineteen cohorts reported these data for NLR analyses. Eight studies directly reported these data for PLR analyses.

**Table 1:** Characteristics of all the studies included in the meta-analysis

Reference, year	Country	Nos.	HR	Design (period)	No of Patients(M/F)	TNM stage	Histology	NLR cut-off	PLR cut-off	Treatment	Prognosticvalue analyses	Survival outcomes
**Youngjoo Lee 2012 [27]**	Korea	6	U/M	Retrospective	199 (17/182)	III–IV	NSCLC	3.25	NR	C	NLR	OS and PFS
**S. Cedré 2012 [28]**	Spain	*7*	U/M	Retrospective	171 (143/28)	IV	NSCLC	5	NR	C	NLR	OS and PFS
**Yanwen Yao 2012 [29]**	China	*7*	U/M	Retrospective	182 (63/119)	III–IV	NSCLC	2.63	NR	C	NLR	OS and PFS
**M H Kang 2014 [22]**	Korea	*8*	U/M	Retrospective	187 (162/25)	I–IV	SCLC	4	160	C	NLR PLR	OS and PFS
**D J Pinato 2014 [9]**	UK	*7*	U/M	Retrospective	220 (110/110)	I–III	NSCLC	5	300	S/C	NLR PLR	OS and PFS
**GuannanWu 2014 [30]**	China	*7*	U/M	Retrospective	366 (246/120)	III–IV	NSCLC	2.68	119.5	NR	NLR PLR	OS and PFS
**Mehmet Kos 2014 [16]**	Turkey	7	U/M	Retrospective	145 (130/15)	I–IV	NSCLC	NR	198.2	S/C	PLR	OS
**Tiehong Zhang 2014 [31]**	China	7	U/M	Retrospective	400 (272/128)	I–II	NSCLC	2.6	200	S	NLR PLR	OS and DFS
**Turgut Kacan 2014 [32]**	Turkey	6	M	Retrospective	299 (270/29)	I–IV	NSCLC	5	NR	NR	NLR	OS
**Gui-Nan LIN 2014 [33]**	China	7	U/M	Retrospective	81 (47/34)	NR	NSCLC	3.5	NR	TKIs	NLR	OS and PFS
**Xinyue Wang 2014 [34]**	China	6	M	Retrospective	114 (89/25)	NR	SCLC	3	NR	S/C	NLR	OS
**Katsuhiko Shimizu 2015 [35]**	Japan	*7*	U/M	Retrospective	334 (213/121)	I–III	NSCLC	2.5	NR	S	NLR	OS and DFS
**Jae Eun Choi 2015 [36]**	USA	7	U/M	Retrospective	1139 (602/537)	I–III	NSCLC	5	NR	S/C	NLR	OS and RFS
**FahriyeTugba Kos 2015 [37]**	Turkey	6	U/M	Retrospective	138 (124/14)	I–IV	NSCLC	3.24	NR	S/C	NLR	OS
**Yusuke Takahashi 2015 [38]**	Japan	7	U/M	Retrospective	361 (114/152)	I–III	NSCLC	2.5	NR	S	NLR	OS and RFS
**Hua Zhang 2015 [39]**	China	8	U	Retrospective	678 (449/229)	NR	NSCLC	2.3	106	S	NLR PLR	OS and DFS
**Hua Zhang 2015(2) [40]**	China	7	U/M	Retrospective	1238 (812/426)	I–III	NSCLC	2.3	NR	S/C	NLR	OS and DFS

Chemotherapy, DFS disease-free survival, HR hazard ratio, NR not reported, NLR neutrophil-to-lymphocyte ratio, OS overall survival, PLR platelet-to-lymphocyte ratio, PFS progression free survival, S surgery, TNM tumor, node, metastasis classification system.

### Associations between NLR or PLR and prognostic

### Significance of lung cancer

**Figure 2:**
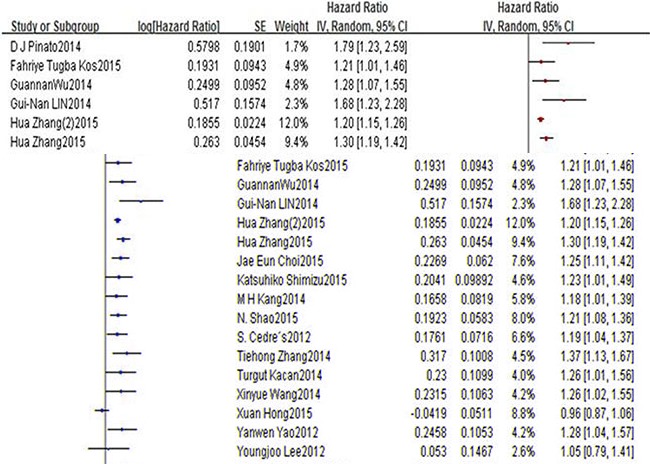
Forest plots of HNLR versus LNLR with OS of all patients in all studies *HNLR* high neutrophil-to-lymphocyte ratio, *LNLR* low neutrophil-to-lymphocyte ratio, *OS* overall survival.

**Figure 3:**
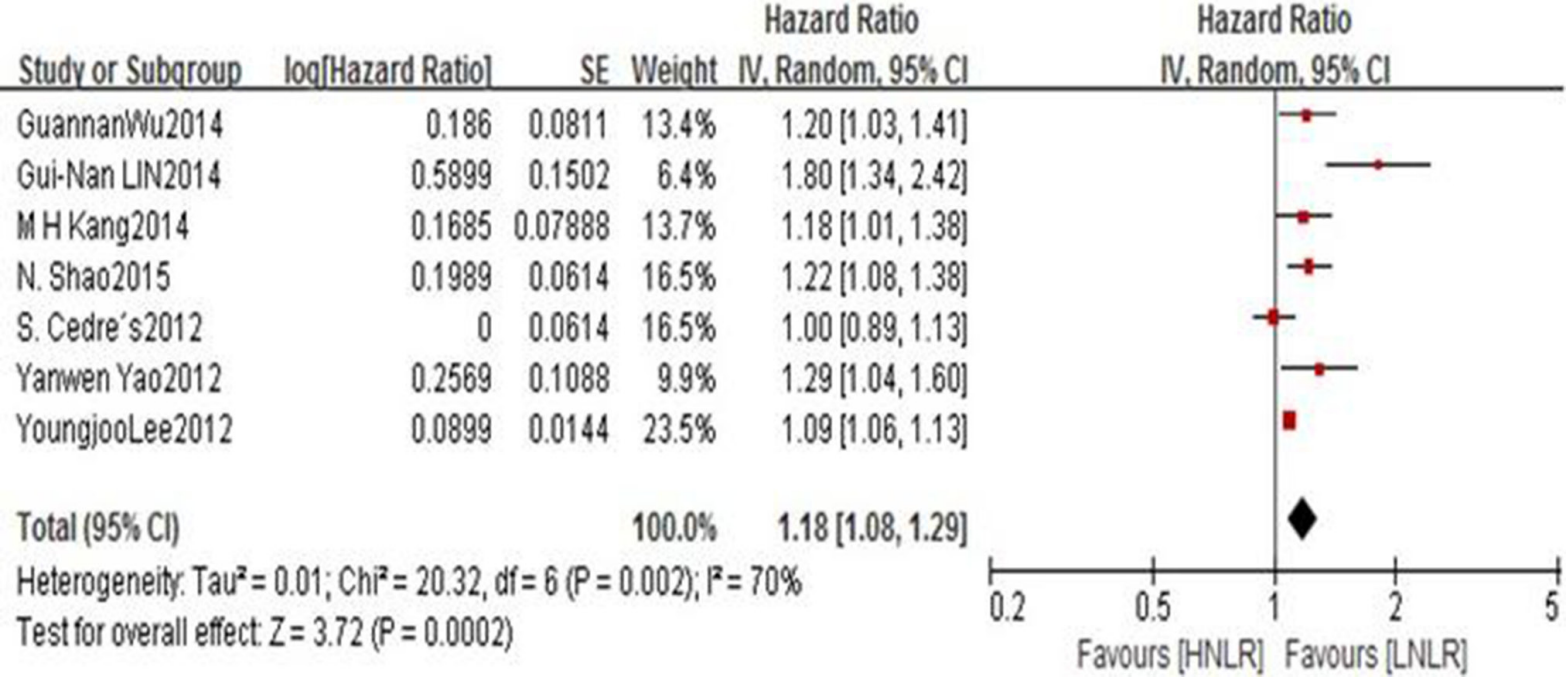
Forest plots of HNLR versus LNLR with PFS of all patients in studies *HNLR* high neutrophil-to-lymphocyte, *LNLR* low neutrophil-to-lymphocyte ratio, *PFS* progression free survival.

Eighteen studies presenting data of 5,881 patients for NLR and OS in lung cancer patients. In both studies, the elevated NLR were expected to have shorter OS with a pooled HR of 1.23 (95% CI: 1.17–1.29; *P* < 0.00001), However, with significant between-study heterogeneity (I^2^ 53%, *P* = 0.004), a forest plot of this is shown as Figure 2. And there showed no significant publication bias by using Egger's test (*P* = 0.075) and Begg's test (*P* = 0.111). There are seven studies which included 1,298 patients investigated the association between NLR and PFS suggested that PFS was significantly poorer in patients with high NLR (HR = 1.18; 95% CI = 1.08 to 1.29, *P* = 0.0002) and heterogeneity was observed (*P* = 0.02, I2 = 70%). Begg's test (*P* = 0.048), but Egger's test (*P* = 0.097), showed no significant publication bias. Figure 3 presentes this analysis by the forest plot. Seven studies presenting data on PLR and OS of lung cancer for estimating HR and 95% CI showed a significant survival in patients with high compared to low PLR (HR 1.07, 95% CI 1.01–1.13, *P* = 0.01), with no significant heterogeneity (I2 = 52%, *P* = 0.05). Begg's test (*P* = 0.133) and Egger's test (*P* = 0.382) also indicated little evidence of publication bias. There is no meta-analysis of PLR and PFS was performed because only two studies reported this data.

### Subgroup and sensitivity analyses

**Figure 4:**
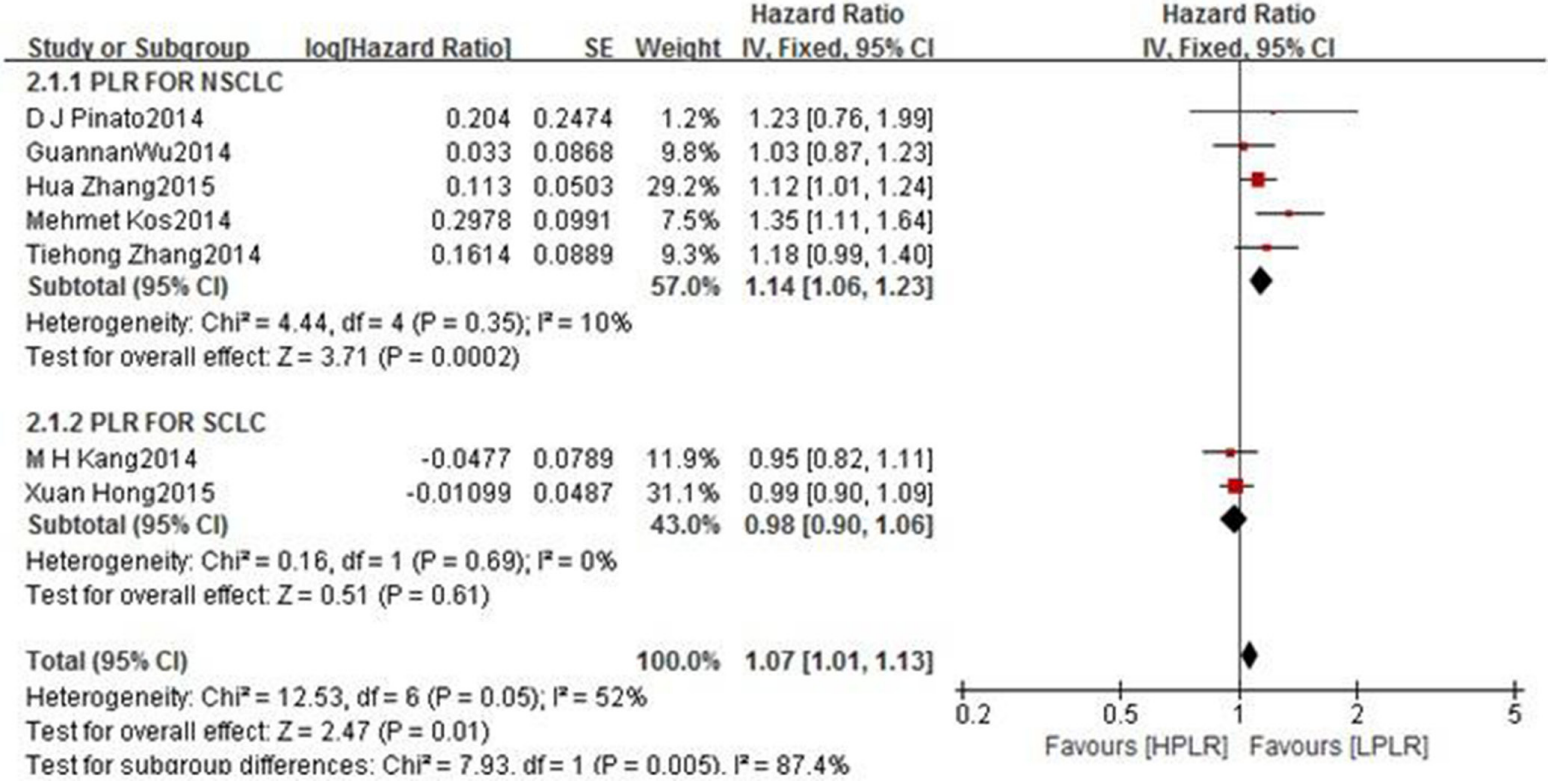
Forest plots of HPLR versus LPLR with OS of all patients in studies and subgroups of patients who are NSCLC or SCLC *HNLR* high neutrophil-to-lymphocyte ratio, *LNLR* low neutrophil-to-lymphocyte ratio *NSCLC* non small cell lung cancer, *OS* overall survival *SCLC* small cell lung cancer.

**Figure 5:**
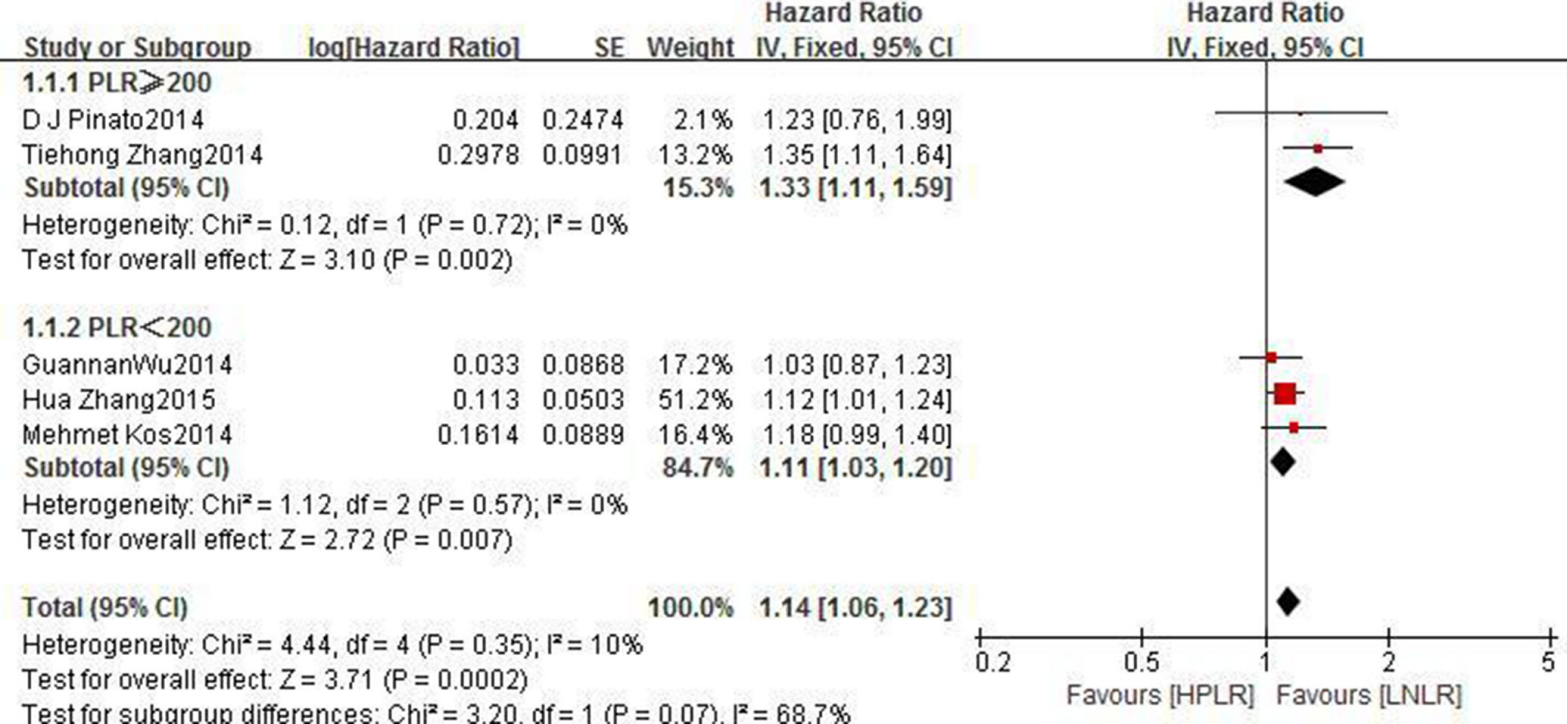
Forest plots of PLR ≥ 200 versus PLR < 200 with OS of NSCLC *HPLR* high platelet-to-lymphocyte ratio, *LPLR* low platelet-to-lymphocyte ratio, *OS* overall survival, *NSCLC* non small cell lung cancer.

The subgroup analyses according to cut-offs for NLR, type of treatment, pathological type and region regarding the effect of NLR on OS is shown in Table 2. The results of most subgroup found little evidence of heterogeneity for high NLR suggested significantly poorer OS. However In subgroup of NLR ≥ 5, the I^2^ = 80%, *p* < 0.00001. From the subgroup which include of PLR on OS in NSCLC, we can got prognostic significance of high PLR (HR 1.14, 95% CI 1.06–1.23, *P* = 0.0002). While, the subgroup of PLR on OS in SCLC has no significance for PLR on OS. (HR 0.98, 95% CI 0.90 – 1.06, *P* = 0.61) (Figure 4). The differences between two subgroups were statistically significant (*P* for subgroup difference = 0.005). From the results above, we made a further subgroup analysis which is about the cut-offs for PLR on OS in NSCLC (Figure 5). It showed the cut-off value of PLR is not the source of heterogeneity (*P* for subgroup Difference = 0.07). Sensitivity analyses investigating the influence by omitting one study at a time and Calculating the combined HRs. Any single study was omitted, the pooled HRS were not substantially affected.

**Table 2:** Subgroup analyses

Subgroup	*N*	HR (95% CI) *P*				*I*^2^ (%)	*P*	P for subgroup difference	
		RE		FE				RE	FE
**Cut-offs for NLR**								0.96	0.05
< 3	7	1.23 (1.19,1.28)	< 0.00001	1.23 (1.19,1.28)	< 0.00001	0%	0.65		
3–4	6	1.22 (1.13,1.32)	< 0.00001	1.22 (1.13,1.32)	< 0.00001	6%	0.38		
NLR ≥ 5	5	1.21 (1.03,1.42)	0.02	1.13 (1.06,1.20)	0.0003	80%	< 0.00001		
**Type of treatment**								0.07	0.07
Chemotherapy	5	1.20 (1.12,1.29)	< 0.00001	1.10 (1.04,1.17)	0.002	0%	0.88		
surgery	5	1.31 (1.23,1.41)	< 0.00001	1.31 (1.23,1.41)	< 0.00001	0%	0.50		
**Pathological type**								0.11	0.03
NSCLC	14	1.24 (1.20,1.28)	< 0.00001	1.24 (1.20,1.28)	< 0.00001	2%	0.43		
SCLC	4	1.15 (1.06,1.25)	0.0010	1.14 (1.07,1.22)	< 0.0001	35%	0.20		
**Region**								0.53	0.50
Easterncountries	15	1.22 (1.17,1.27)	< 0.00001	1.21 (1.18,1.25)	< 0.00001	23%	0.20		
Westren countries	3	1.28 (1.11,1.48))	0.0006	1.25 (1.15,1.37)	< 0.00001	49%	0.14		

FE fixed-effect model, HR hazard ratio, RE random-effect model, NSCLC non small cell lung cancer, N number, NLR neutrophil-to-lymphocyte ratio, SCLC small cell lung cancer.

### Publication bias

There was no evidence of publication bias because of bias exploration funnel plots demonstrated symmetry. Egger's test and Begg's test also validates little publication bias. The associations between NLR and Clinicopathologic Parameters. For the associations between NLR and Clinicopathologic Parameters which are summarized in Table 3, showed that high NLR was significantly associated with deeper Invasive of tumor, (OR 1.54,95% CI 1.13–2.09, *P* = 0.006) extensive lymph node metastasis(N2-3)(OR 1.47,95% CI 1.09–1.97, *P* = 0.01), poor differentiation(OR1.72,95% CI 1.30 – 2.29, *P* = 0.0002))and vascular invasion(OR1.70,95% CI 1.21 – 2.40, *P* = 0.002). But it has no evidence to support that high NLR was associated with worse tumor stage (OR 0.92,95% CI 0.65–1.32, *P* = 0.66). The heterogeneity of all studies has no significance with *P* > 0.05.

**Table 3:** Associations between NLR and clinicopathologic parameters

Parameter	Study no. [references]	No. of patients	OR (95% CI, *P*)	*I*^2^ (*P*)
Invasive tumor (T3–4)	3	719	1.54 (1.13–2.09,0.006)	16% (0.30)
Lymph node Metastasis) (N2-3)	4	1243	1.47 (1.09–1.97,0.01)	0% (0.56)
Poor differentiation	4	1053	1.72 (1.30–2.29,0.0002)	0% (0.71)
Vascular invasion	2	695	1.70 (1.21–2.40,0.002)	61% (0.11)
Tumor Stage (IV)	3	686	0.92 (0.65–1.32,0.66)	61% (0.08)

OR odds ratio.

## DISCUSSION

The systematic inflammatory response of cancer-related can be easily embodied by measuring available blood parameters such as NLR, PLR [9]. NLR which is one of markers of systemic inflammation was accepted to be associated with prognosis in different cancers. NLR may present the pro-angiogenic/pro-inflammatory status in tumor tissue [10], and may also show how to balance neutrophils and lymphocytes, and then reflect patients' immune function [11, 12]. Preclinical studies showed that neutrophils may act through TGF-β induced signal pathway, with tumor promoting proliferation of leucocytes [13]. Patients with an elevated NLR exhibit the ratio of neutrophils and lymphocytes and may indirectly suggests poorer lymphocyte-mediated immune response to tumors, therefore, accelerating the process of tumor and prompting with worse prognosis [14]. Cytokines, such as vascular endothelial growth factor (VEGF) and transforming growth factor β, are meaningful in tumor angiogenesis. Platelets were also considered to be the major sources of these cytokines. PLTs could be elevated because of tumors or inflammatory cells releasing inflammatory mediators which can stimulate megakaryocyte release platelets [15]. PLR are reported that is associated with poor prognosis in many kinds of malignant tumors such as the pancreas, esophagus, stomach cancers in many studies [16].

Changes of the NLR and PLR levels has been reported to be predictive markers were consistent with chemotherapeutic efficacy and prognosis, and so NLR and PLR associated with pathological response to neoadjuvant chemotherapy or preoperative chemoradiotherapy in gastric cancer, esophageal and rectal cancers [17–19]. In our subgroup analysis of treatment, as for the NLR’ prognostic significance, there is no difference in surgery or chemotherapy. And we can not highlight NLR's prominent predictiverole in the chemotherapy. So it's needs more studies to clarify NLR and chemotherapy prognostication in lung cancers.

The present meta-analysis of 19 studies comprising 7,283 patients with lung cancers provides further evidence that high NLR was associated with poorer prognostic significance for lung cancer. High NLR compared with the low could predict OS and PFS in patients. In our subgroup analysis and sensitivity analyses associations did not significantly modified by type of treatment, pathological type and region. In addition, it showed the cut – offs of NLR < 5 were statistically worse affected than the patients with the cut – offs of NLR ≥ 5. Furthermore, elevated PLR could also predict OS of lung cancer patients. But, in subgroup analysis, as for SCLC, high PLR was not associated with a poorer prognosis.

All the intrinsic characteristics of tumor cells and the tumor microenvironment can be act to tumor progression and metastasis, which is mainly influenced by inflammatory cells, including neutrophile granulocyte [20].

Immune system promotes tumour vessel regeneration, migration, invasion, and metastasis by raising regulatory T lymphocytes and activation of related modulators such as IL-6 and TNF-α, C reactive protein, induction of neutrophilia, battering down immune system [21]. In our study there was also a significant association between NLR and Clinicopathologic Parameters, such as deeper Invasive of tumor, extensive lymph node metastasis, poor differentiation and vascular invasion. Taking all these into consideration, NLR and PLR are useful prognostic indicator in lung cancer. There are some important strengths in our study. Compared with the previous studies, we have larger sample size and with evidence to provide the association between high NLR and poor Clinicopathologic Parameters. Potential limitations should be concerned too. First we could not exclude uncontrolled or unmeasured risk factors from original studies that have confounded the true association. Second, Small-cell lung cancer (SCLC) accounts for 15–20% of all lung.

cancers and has an overbearing nature with a poor prognosis [22]. The studies we collected was rarely describe the PLR, furthermore the lack of studies showing the relationship between PLR and clinical pathological data. Third all articles are in English and the heterogeneity of some research is relatively large, which may be caused by some unmeasured factors. Finally, platelet, neutrophil and lymphocyte counts would be affected by the patients' basic state, infection, chemotherapy and other related factors [23], the subgroup proved that the heterogeneity of treatment is not significant. But we can not rule out the existence of patients' own inflammatory conditions completely.

## MATERIALS AND METHODS

### Search strategy

We attempted to report this meta-analysis in accordance with the Meta-Analysis of Observational Studies in Epidemiology guidelines. We conducted a systematic literature search of the PubMed database, Google Scholar, Cochrane databases and Springer link up to December 2015 by using the following search terms. NLR” (or “neutrophil lymphocyte ratio,”) OR “PLR”(or “platelet lymphocyte ratio,”) AND “lung cancer” AND “survival” Reference lists of the retrieved articles were also reviewed. We did not contact authors of the primary studies for additional information.

### Inclusion and exclusion criteria

Study selection was based on an initial screen of identified abstracts or titles and a second screen of full-text articles. Studies were included if they met the following criteria:1) studies had to compare survival outcomes in lung cancer patients with high NLR (or PLR) versus low NLR (OR PLR) and report their cut-off values, and 2) availability of a hazard ratio(HR) and 95% confidence interval (CI) or a *P* value for overall survival (OS) or progression-free survival (PFS). Exclusion criteria were: (1) the full text was not available of quality assessment and data extraction; (2) abstracts, letters, editorials, expert, case reports or review articles; and (3) non-clinical studies or case reports.

### Data extraction and quality assessment

All potentially eligible studies reviewed by two investigators independently and they collected data of patients and study characteristics. Conflicts of Data extraction or quality assessment were resolved by discussion and consensus. The inclusion/exclusion criteria and outcome measures are described below. (Table 1)The Newcastle-Ottawa Scale (NOS) was used to assess study quality. The NOS consists of three parameters of quality: selection (0–4 points), comparability (0–2 points), and outcome assessment (0–3 points). Studies with scores of 6 representing the high quality methodological study. OS and PFS were the primary outcomes of interest. The following details were extracted: (first author, year of publication, country of origin, number of patients included in analysis, disease Stage, histologic type, tumor invasion, lymph node status, metastasis, cut-off defining of peripheral blood NLR (or PLR), and hazard ratios and associated 95% confidence intervals for OS, PFS as applicable. Hazard ratios were extracted preferentially from multivariable analyses where available. If not, we extract hazard ratios from univariate analyses.

### Statistical analyses

Extracted data were combined into a meta-analysis using RevMan5.3 analysis software. The primary summary is logarithm of the hazard ratio (HR) with 95% confidence interval (CI) [24]. The data of HR and 95% CIs were obtained directly from individual articles or were calculated from indirect data [24]. Practical methods for incorporating summary time-to-event data into meta-analysis. Meta-analysis of the data was conducted using random-effects model and the fixed-effects model. Publication bias was explored graphically by visual inspection of funnel plots to detect asymmetry and any outliers and was assessed by Egger's *I* test and Begg's test. When *P* < 0.05, they were considered to be significantly biased [25, 26]. Heterogeneity was assessed using the square statistic and the *I*^2^ value which is a quantitative measure of in consistency across studies. This was graded as low (*I*^2^ < 25%), moderate (*I*^2^ = 25 to 75%) or high (*I*^2^ > 75%). and was considered significant at the *P* < 0.05 level. We conducted subgroup analyses according to cut-offs for NLR, type of treatment, pathological type and region. All statistical tests were two-sided, *P* value < 0.05 was considered statistically significant, except where otherwise specified.

## CONCLUSIONS

This meta-analysis suggests that the high NLR and PLR is associated with worse survival in lung cancer patients and support a significantly relation between high NLR and deeper Invasive of tumor, extensive lymph node metastasis, poor differentiation and vascular invasion. However, there needs more well-designed and large-scale studies to demonstrate the PLR's value in SCLC and the connection of high PLR with clinicopathologic parameters. High systemic inflammation as measured by NLR and PLR can well assess the poor survival among lung cancer patients in clinical application.
